# Greenness, civil environment, and pregnancy outcomes: perspectives with a systematic review and meta-analysis

**DOI:** 10.1186/s12940-020-00649-z

**Published:** 2020-08-27

**Authors:** Kyung Ju Lee, Hyemi Moon, Hyo Ri Yun, Eun Lyeong Park, Ae Ran Park, Hijeong Choi, Kwan Hong, Juneyoung Lee

**Affiliations:** 1 Department of Obstetrics and Gynecology, Korea University Medicine, 73, Goryeodae-ro, Seongbuk-gu, Seoul, 02841 South Korea; 2 Department of Preventive Medicine, Korea University College of Medicine, Seoul, South Korea; 3 Department of Public Health, Korea University Graduate School, 73, Goryeodae-ro, Seongbuk-gu, Seoul, 02841 South Korea; 4 Department of Biostatistics, Korea University College of Medicine, 73, Goryeodae-ro, Seongbuk-gu, Seoul, South Korea; 5 Graduate School of Integrative Medicine, Cha University, Pocheon-si, Gyeonggi-do South Korea

**Keywords:** Greenness, Green space, Air pollution, Civilization, Pregnancy outcomes

## Abstract

**Background:**

Various maternal conditions, especially in utero conditions and prenatal exposure to environments with air pollution and greenness, have been reviewed to address the enhancement and prevention of susceptibility to health risks, including low birthweight, preterm delivery, and preeclampsia. This study aimed to qualitatively and quantitatively investigate the associations between pregnancy outcomes and the characteristics of surrounding living environment, including greenness, air pollution, and civilization.

**Methods:**

A secondary search of the MEDLINE, EMBASE, Cochrane Library, K-eArticles, and CINAHL databases was conducted without language restrictions to identify the relevant publications from the time of inception of the databases to April 2019.

**Results:**

A total of 89 studies were identified, and 10 were included in the quantitative synthesis. The greenness of the environment within 100-, 250- and 500-m buffers, after adjusting for the air quality and civilization factors, was weakly but positively associated with birthweight. The pooled regression slope was 0.00134 (95% confidence interval [CI], 0.000, 0.0020). The greenness of the environment was also associated with a significant decrease in the incidence of poor pregnancy outcomes, namely, low birthweight, small for gestational age (odds ratio [OR] 0.94; 95% CI, 0.92, 0.97), and preterm delivery (OR 0.98; 95% CI, 0.97, 0.99).

**Conclusions:**

The greenness of the environment had a positive effect on the pregnancy outcomes, despite poor air quality and civilization. Following urbanization, planning for greenness management, environmental medicine, and public health is important and thus should be proposed as preventive methods as way of increasing birthweight and life expectancy.

## Background

Recently, the association between environmental factors and birth outcomes have been reviewed to address the effect the environmental factors before, during, and after pregnancy [1, 2]. The fraction of the global burden of disease due to the environment is estimated to be up to 22% [3] and the urbanization of the environment associated with air pollution is also gradually increasing worldwide [4]. Although not fully understood, greenness in the urban environment is drawing attention as a potential health benefit for everyday life including mental and physical activities [2, 5–7]. In general, greenness is considered to reduce exposure to heat, noise, and air pollution and has a therapeutic effect on mental health [6, 8]. Greenness is believed to primarily strengthen mental and physical activity, reduce mental and physiological stress, and support the public health community [8–10].

The Barker hypothesis, proposed in 1990, states that early intervention during pregnancy improves the health of newborns, which is greatly affected by various maternal conditions, including but not limited to nutrition, obesity, and gestational diabetes mellitus [11]. Exposure of pregnant women to ambient air pollution and to gradually increasing urbanization has been associated with adverse pregnancy outcomes such as low birthweight (LBW), preterm birth, intrauterine growth restriction, and congenital anomalies [12–15]. Pregnancy health outcomes associated with greenery were positively related to desirable pregnancy outcomes including increased birthweight and decreased preeclampsia and postpartum depression [16–20]. Herein, we aimed to investigate the associations between pregnancy outcomes and the characteristics of surrounding living environment, especially greenness, air quality, and civilization. Our research question was “What is the effect of greenness on maternal and neonatal health in the context of air pollution and related civilization?”

## Methods

This systematic review and meta-analysis were performed in accordance with the Preferred Reporting Items for Systematic Review and Meta-analyses statement [21].

### Data sources

The MEDLINE, PubMed, EMBASE, Cochrane Library, Korean Studies Information Service System (K-eArticles), and CINAHL databases were searched for reports published up to April 2019 without language restrictions.

### Search strategy

A PubMed search for studies on green spaces, birth, or pregnancy outcomes was conducted without restrictions by combining search terms related to or synonymous with these terms. The keywords used in the PubMed search were “pregnant woman” or “pregnancy” for population; “green space,” “park,” “forest,” or “greenness” for intervention; and “pregnancy outcome” or “birth outcome” for outcome. The terms were then converted to search tags that were used for the Cochrane Library and EMBASE database searches. For the “birth outcome,” we included search terms suspected to be affected by the environment, such as “maternal illness,” “birthweight,” and “preterm birth” (Supplementary Table 1). Furthermore, the reference lists of the relevant articles were manually searched to identify additional studies.

### Study selection

Greenness is an environmental characteristic, and access to green space is often not equitably distributed with respect to the socioeconomic backgrounds of the city residents. The normalized difference vegetation index (NDVI) is commonly used as an indicator of the presence and level of greenness. The NDVI was defined as the greenness measurement derived from the Landsat Enhanced Thematic Mapper Plus (ETM+) data at a resolution of 30 by 30 m. Landscape databases such as Google Maps or the Greenspace Map in Scotland, which are adjusted for the effects of water-related variables, were excluded in this present study. The overall NDVI on the study-related population was also excluded. We selected buffer sizes of 100, 250, and 500 m.

The exclusion criteria for the studies were as follows: absence of original data, such as that noted in review articles, editorials, commentaries, letters without new data analyses, meta-analyses, and abstracts only; greenness not presented as NDVI but as other parameters, such as percentage of green space in each urban or rural census area unit (CAU), tree-canopy cover, amount of total natural space; absence of the outcomes of interest in cases of gestational diabetes, gestational hypertension, preeclampsia; incorrect publication type; duplicated publication; and inconsistent variations in methodology.

### Data extraction

The extracted information included the family name of the first author, year of publication, country of origin, number of study subjects, study duration, buffer size investigated, covariates used as cofounders for adjusting in a statistical model, outcomes assessed (birthweight, LBW, very LBW [VLBW], small for gestational age [SGA], preterm delivery [PTD], and/or very preterm delivery [VPTD]). In the present study, birthweight was defined as follows: The term birthweight was applicable to full-term births and was defined as term-appropriate birth weight of > 10% of the birthweight at > 37 weeks of gestational age. Epidemiologically various estimated SGA based on the reference population SGA was defined as birthweight < 10% of that for a specific gestational age. Preterm birth was defined as birth before completion of 37 weeks of gestation. Low birthweight (LBW) and very LBW (VLBW) were defined as birthweight < 2500 g and < 1500 g, respectively.

The covariates were categorized into subject demographic variables; those related to air pollution such as NO_x_ and/or particulate matter (PM)_x_; and factors regarding civil environments such as population density, noise, and/or distance to the nearest park.

Most studies employed a multivariable linear regression or nonlinear regression spline models to evaluate the effects of greenness on birthweight and multivariable logistic regression or generalized estimating equation models to examine its effect on the odds of (V) LBW, SGA, or (V)PTD. Therefore, as effect sizes, we used the reported regression coefficients and the adjusted odds ratios, along with their standard errors.

We investigated the greatest possible impact of greenness when we extracted the estimated adjustment effect with standard error or the corresponding 95% confidence intervals (CIs). We selected a model that reflected the greatest potential confounders for the key analysis that was provided in the body of the article or the supplementary material. The characteristics of the studies are shown in Table 1 according to the NDVI buffer size, covariates, and pregnancy outcomes.

**Table 1:** Characteristics of the included studies regarding the effects of the NDVI on birth weight and risk of low birth weight or preterm birth

Author	Publication year	Country (region)	Number of subjects	Study period	Buffer size examined (m)	Adjustment factors			Definition of birth weight	Outcome	Multivariable model used	NDVI exposure units^a^	Effect size calculation for meta-analysis ^a^
						Subject demographics	Air quality	Civilization factor					
Cusack et al. [22]	2017	US (Austin)	88,807	2005–2009	50, 250, 500, 1000	Mother’s and father’s age, smoking, gestational age, baby’s sex, year, month, mother’s and father’s education, prenatal care, parity, race/ethnicity, household income, % without high school education, unemployment, % below poverty, % white, % Hispanic	NO_2_	Population density	Birthweight for full term babies (≥37 weeks of gestation)	Birthweight	Stratified multivariable linear regression model for outcome (birthweight)	0.1	B → β
		US (Portland)	90,265										SD of NDIV was imputed using its IQR
Agay-Shay et al. [23]	2014	Israel	39,134	2000–2006	100, 250, 500	Maternal origin of birth, infant sex, infant religion, marital status, season of conception, maternal age, year of birth, ward-based socioecological status, week of gestation			Term birthweight (gestational age at delivery ≥37 weeks)	Birth weight, LBW, VLBW and SGA, PTD and VPTD	Multivariable linear model for (birthweight) / multivariable logistic regression model for (LBW, VLBW and SGA), (PTD and VPTD)	IQR	B → β
Laurent et al. [24]	2013	US	81,186	1997–2006	50, 100, 150	Maternal age and maternal age squared, length of gestation and length of gestation squared, poverty and poverty squared, insurance status, race/ethnicity, parity, diabetes, and infant’s sex	NO_x_ or PM_10_ or PM_2.5_ orLocal traffic-generated NO_x_	Traffic density within 50 m	Infants born at term (≥37 weeks of gestation)	Birth weight, PTD, and VPTD	GEE (generalized estimation equations) for (birthweight), (PTD and VPTD)	IQR	B → β
													SD of NDIV is imputed using its IQR SD of birth weight is imputed ^b)^
Glazer et al. [25]	2018	US	56,633	2001–2012	150, 250, 500	mother’s age, prenatal visits, tobacco use, parity, education, race, insurance, marital status, gestational age, neighborhood SES		Town of residence (such as access to medical care and walkability)	Term birth weight	Birth weight, LBW, VLBW and SGA; PTD and VPTD	Multivariable linear model for (birthweight) / multivariable logistic regression model for (LBW, VLBW, and SGA), (PTD and VPTD)	IQR	B → β
													SD of NDIV for 250 m buffer size is imputed by that of 500 m buffer size
Hystad et al. [26]	2014	Canada	64,705	1999–2002	100	Sex, parity, First Nations status, maternal age, maternal smoking during pregnancy, maternal education, income, year and month of birth, completed weeks of gestation	NO, NO_2_, PM_2.5_, BC	Noise, Walkability/parks	Term births weight (≥37 weeks of gestation)	Birth weight, LBW, VLBW, and SGA, PTD and VPTD	Multivariable linear model for (birthweight) / multivariable logistic regression model for (LBW, VLBW, and SGA), (PTD and VPTD)	IQR	B → β
Fong et al. [27]	2018	US (0.25–0.50 NDVI)	780,435	2001–2013	250	Maternal age, race, smoking before or during pregnancy, education, parity, chronic diabetes, gestational diabetes, chronic high blood pressure, gestational high blood pressure, Kessner index of adequacy of prenatal care, birth mode of delivery, clinical gestational age, newborn sex, government support for prenatal care, season of birth, year of birth, proportion census black population, Census median household income	PM_2.5_	Population density	Full-term births (≥37 weeks gestation)	Birth weight, LBW, VLBW, and SGA	Nonlinear (logistic) model with a natural spline for (birth weight), (LBW, VLBW, and SGA)	0.1	B → β
		US (0.50–0.75 NDVI)											
Markevych et al. [28]	2014	Germany	3203	1996–1999	100, 250, 500, 800	Study, year of birth, season of birth, sex, maternal age, maternal education level, maternal smoking during pregnancy	NO_2_ or PM_2.5_	Proximity to the major road or population density	Full-term neonates (gestational age ≥ 37 weeks) with a normal birth weight (> 2500 g)	Birth weight	Multivariable linear regression model	IQR	B → β
Dadvand et al. [16]	2012	Spain	2393	2003–2008	100, 250, 500	Gestational age, maternal age, ethnicity, socioeconomic status, education level, pregestational BMI, weight gain during pregnancy, smoking, alcohol consumption, parity, sex of infant, paternal BMI, season of conception	NO_2_		Term births (gestational age at delivery ≥37 weeks)	Birth weight	Linear mixed models with a random intercept	IQR	B → β
													SD of NDIV is imputed using its IQR
Grazuleviciene et al. [20]	2015	Lithuania	3292	2007–2009	100, 300, 500	Maternal and paternal height, maternal active smoking, marital status, infant sex, gestation duration, parity, BMI, previous preterm birth, maternal diabetes and chronic hearth diseases				LBW, VLBW, and SGA; PTD and VPTD	Multivariable linear model / multivariable logistic regression model for (LBW, VLBW and SGA), (PTD and VPTD)	IQR	B → β
Cusack [29]	2018	Canada	2510	2009–2012	100, 250, 500, 1000	Gestational age, infant sex, year and month of birth, mother’s age, mothers smoking during pregnancy, mother/father education, mother/father race/ ethnicity, household income, indoor air quality index, and city	NO_2_, PM_2.5_, O_3_	Population density at 1 km	Term birth weight (≥37 weeks of gestation)	Birth weight	Linear mixed models with a random intercept for (birthweight)	IQR	B → β
													SD of NDIV is imputed using its IQR ^a)^

^a^*Abbreviations*: *IQR* Interquartile range, *B* Unstandardized adjusted regression coefficient, β Standardized adjusted regression coefficient, *SD* Standard deviation; *IQR* Interquartile range, *LBW* Low birth weight, *VLBW* Very low birth weight, *SGA* Small for gestational age, *PTD* Preterm delivery, *VPTD* Very preterm delivery, *BMI* Body mass index

The SD of NDVI for 100- and 250-m buffer distance was imputed by the IQR of the 500-m buffer distance

The SD of the birthweight was imputed as the average SD of other studies

### Data and statistical analyses

As the buffer sizes of the NDVI examined varied among the included studies, we selected buffer sizes of 100, 250, and 500 m for which at least two studies were available. The outcomes of our quantitative synthesis were summarized as (1) term birthweight, (2) low birthweight-related outcomes (LBW, VLBW, or SGA), and (3) preterm birth (PTD or VPTD). The confounding factors for the effect of the NDVI on these outcomes were categorized as subject demographic characteristics, factors associated with air quality, and factors associated with civilization. The degree of civilization was mainly expressed by the population density and traffic density, town of residence, noise, or walkability.

The estimated regression coefficient and its standard error for the effect of greenery on the birthweight from the results of multivariable regression models were categorized according to the buffer size and types of confounders. In this review, owing to the different NDVI exposure units, a standardized regression coefficient for birthweight was used as an effect size index [30]. The standardized coefficient was calculated using the following formula: [(SD of NDVI)/(SD of birthweight) × unstandardized coefficient] [31]. When the SD of the NDVI was not reported, we imputed it using the following formula: (third quartile of the NDVI –first quartile of the NDVI)/1.349, if the interquartile ranges were provided for the NDVI or (maximum NDVI –minimum NDVI)/6, if the NDVI range was reported. A standardized odds ratio (OR) for the effects of greenness on pregnancy outcomes (low birthweight-related outcomes or preterm birth) was also calculated as follows: (natural logarithm of the reported OR divided by the reported NDVI exposure unit is multiplied by 0.1 and then exponentiated.

A meta-analysis was conducted using a random-effects model owing to the limited number of studies available along with the observed heterogeneity among the studies. The synthesized results were presented as standardized regression coefficients or standardized ORs, as appropriate, with corresponding 95% CIs. The statistical heterogeneity between studies was assessed using the tau-squared values, Cochran’s Q-tests, and Higgins’ I-squared statistics. For the Q statistic, heterogeneity was considered to be present if the *P*-value was < 0.1. We defined low, moderate, and high heterogeneity as I^2^ values of 25, 50, and 75%, respectively.

Publication bias was evaluated by visual inspection of the contour-enhanced funnel plots [23, 32] and was tested using Egger’s tests [33]; P-value of < 0.1 was considered indicative of publication bias. No subgroup analysis was pre-defined and performed.

However, sensitivity analyses were conducted for the effect of greenness on the term birthweight by omitting the most influential study [34] and by restricting studies that adjusted for the PM_x_ of the air quality and demographic characteristics of the subjects [35, 36] to test the robustness of the overall pooled results. In addition, random-effects meta-regression analysis was performed to examine the dose-response relationship between the effects of buffer size and NDVI on birthweight.

All statistical analyses were conducted using R version 3.6.0 (R Foundation for Statistical Computing, Vienna, Austria) and Review Manager version 5.3 (The Cochrane Collaboration, The Nordic Cochrane Centre, Copenhagen, Denmark). Stat *P*-values < 0.05 were considered statistically significant, except for the results of the Cochran’s Q-test and in the evaluation of publication bias.

### Quality assessment

The Risk of Bias Assessment tool for Non-randomized Study (RoBANS) was used to assess the risk of bias in the included studies [37]. The RoBANS consists of six domains that include participant selection, confounding variables, intervention measurement, outcome assessment blinding, incomplete outcome data, and selective outcome reporting. The tool assessed the risk of bias for each domain as high, low, and uncertain.

## Results

**Fig. 1:**
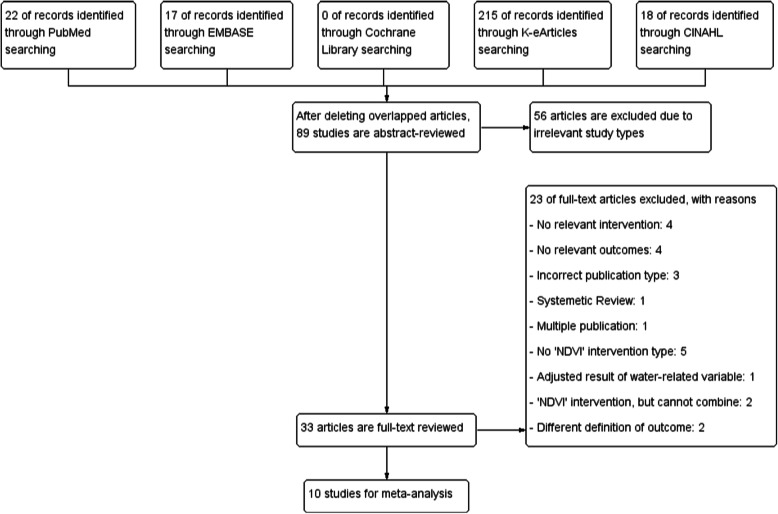
Flow diagram of the search strategy and study selection

**Fig. 2:**
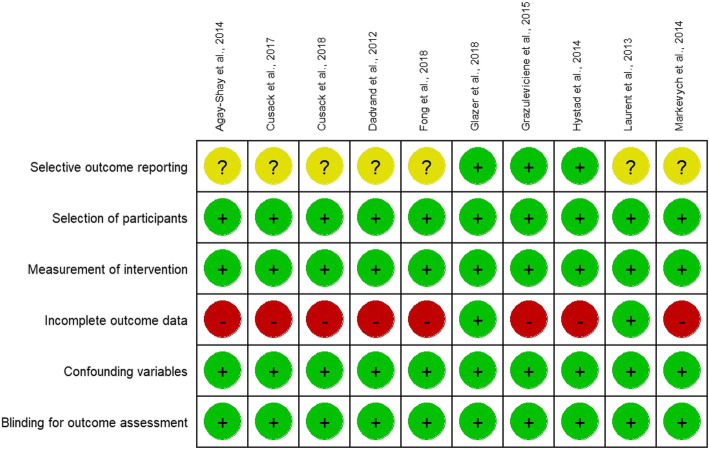
Risk of bias summary. The authors’ judgments regarding each risk of bias item for the included studies were determined using the Risk of Bias Assessment tool for Non-randomized Study

The study selection process is detailed in Fig. 1. Briefly, after an initial screening of the titles and abstracts of 89 potentially relevant articles on the effect of the NDVI for greenness on birth or pregnancy outcomes, the full text of 33 studies was reviewed. After excluding 23 studies that did not meet the inclusion criteria, we identified 10 articles (9 articles for birthweight [20, 22, 24, 28, 34–36, 38, 39]; 5 articles for LBW [20, 22, 28, 29, 39]; and 5 articles for preterm birth [20, 22, 29, 35, 39]). The characteristics of the included studies are summarized in Table 1 and the risk of bias is shown in Fig. 2. All included studies were published in 2012 or later, had sample sizes ranging from 2393 to 780,435 participants, and study years ranging from 1996 to 2013. Our studies included a total of 1,212,563 subjects. All studies were performed in North America or Western Europe, except for one from Israel and one from Lithuania. The examined buffer sizes varied among studies, from 50 to 1000 m but most included 100, 250, and 500 m. Analysis of air pollution also varied from NO, NO_2_, and O_3_ to PM_2.5_ and PM_10_.

To assess the effect of greenness on term birthweight after adjusting for potential confounders, seven of the 10 studies included in the meta-analysis employed a multiple linear regression model, whereas three studies [27, 28, 35] used a multiple linear regression model with a random intercept, a multiple linear mixed model with a random cohort effect, and a generalized estimating equation to evaluate within-hospital correlation, respectively.

The 10 studies evaluated the effects of greenness as follows: with adjustment for the subject demographic variables only [20, 22, 24, 34, 35, 39], with adjustment for demographic and air quality variables [22, 34–36], and with adjustment for demographic and civilization factors regardless of air quality management [22, 24, 28, 35, 36, 38, 39].

**Fig. 3:**
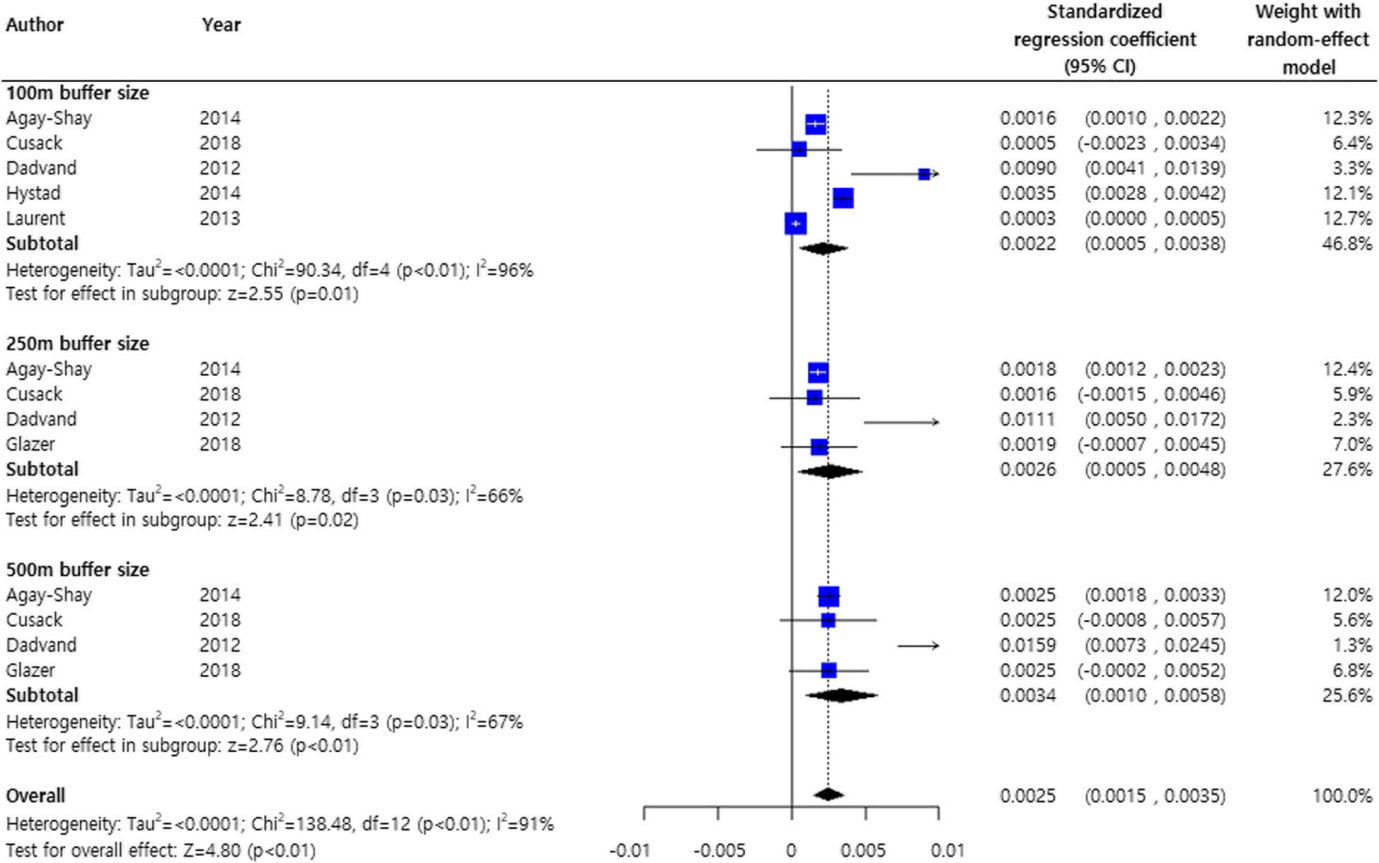
Meta-analysis of standardized regression coefficients of the effects of greenness on term birthweight. The analysis was adjusted for demographic characteristics of the subjects only

Figure 3 shows the results of the meta-analysis on the effects of greenness on birthweight stratified by the NDVI buffer size with standardized regression coefficients and adjusted for demographic variables. Although the heterogeneity among studies was considerable (I^2^ = 91%), the NDVI distance was significantly positively associated with birthweight gain [34]. Except for a very study reporting a high association (Dadvand, et al., 2012c) [16], the overall effect of greenness on birthweight remained significant. (pooled estimate, 0.0019; 95% CI, 0.0009, 0.0028) (Supplementary Figure S1)

**Fig. 4:**
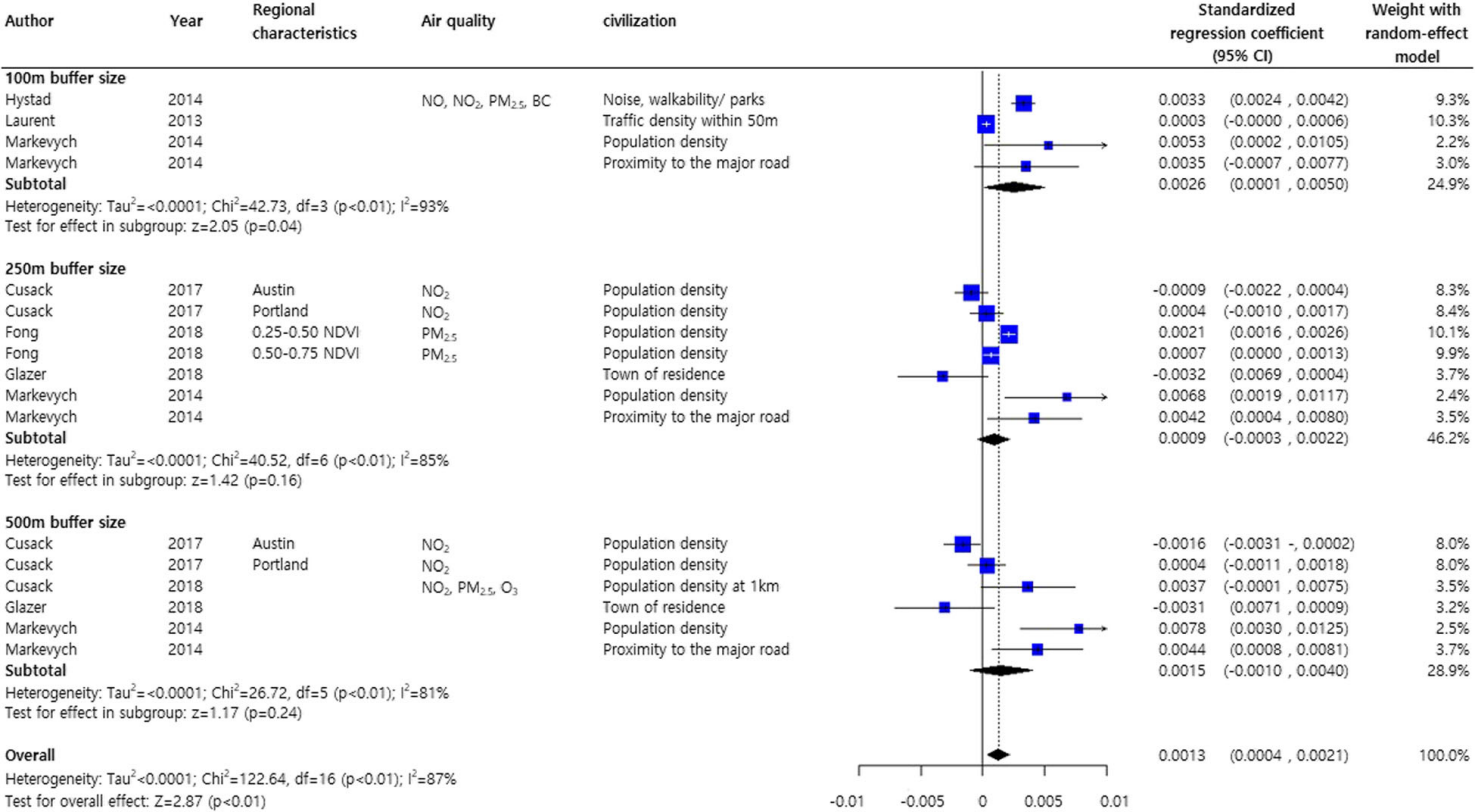
Meta-analysis of standardized regression coefficients for the effects of greenness on birthweight. The analysis was adjusted for subject demographic characteristics and civilization factors regardless of air quality effects

In two studies on the effects of greenness on birthweight stratified by the NDVI buffer sizes (250 and 500 m), a meta-analysis was conducted by adjusting for other air quality factors including PM_2.5_ and PM_10_ [35, 36]. Even in the presence of air pollutants, greenery was associated with birthweight gain (pooled estimate, 0.0003; 95% CI, 0.0002, 0.0005) (Supplementary Figure S2). After adjusting for civilization factors, regardless of air quality, the NDVI buffer distances produced different results for birthweight gain: high birthweight gains with a 100-m buffer distance (0.0026 g, 95% CI, 0.0001, 0.0050), medium birthweight gain with a 500-m buffer distance (0.0015 g, 95% CI, − 0.0010, 0.0040), and LBW gain with a 250-m buffer distance (0.0009 g, 95% CI, − 0.0003, 0.0022) (Fig. 4).

**Fig. 5:**
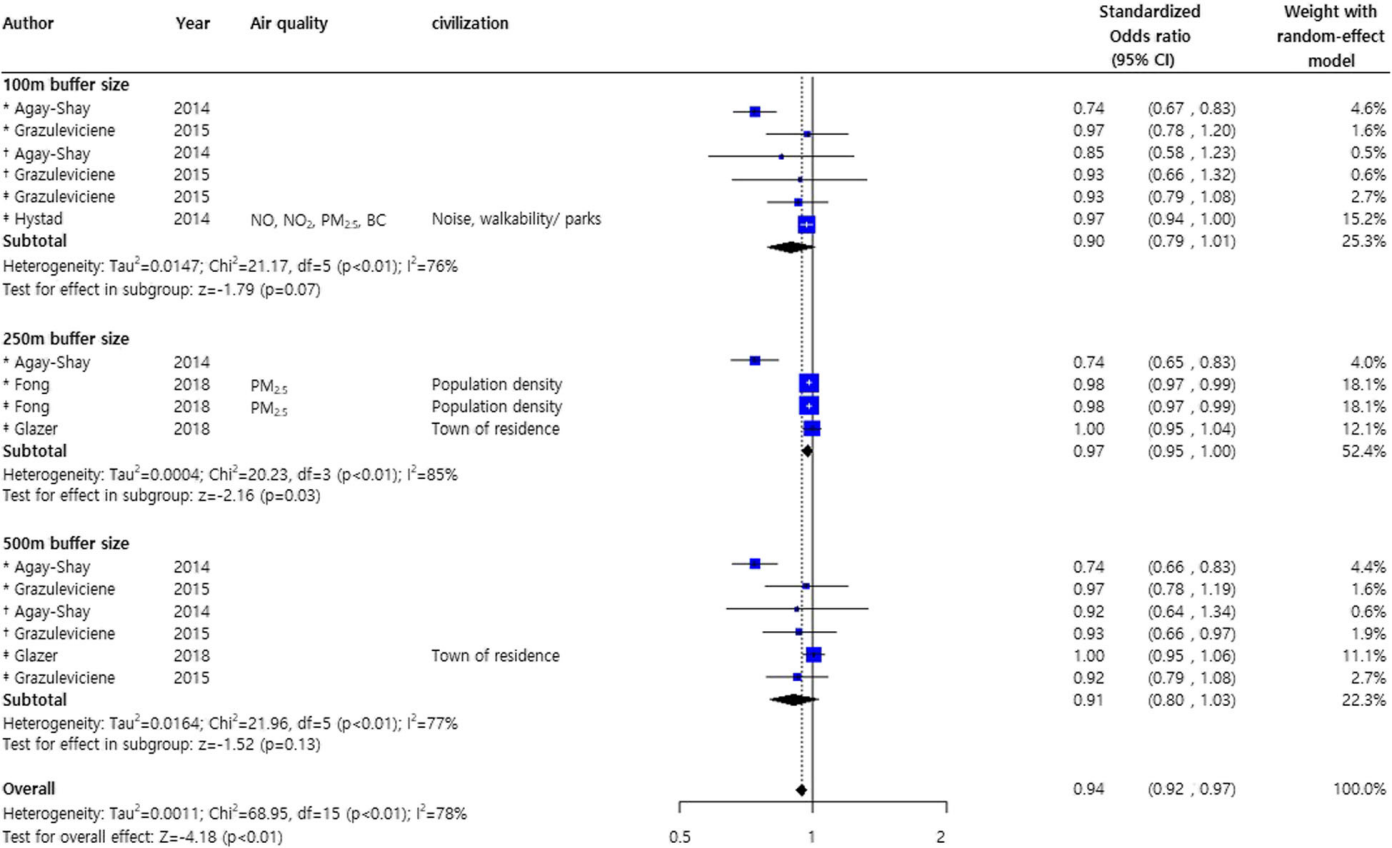
Forest plot for the effects of greenness on low birthweight*, very low birthweight †, and small for gestational age ‡.Abbreviations: LBW: low birthweight; VLBW: very low birthweight; SGA: small for gestational age

Investigation of the effects of greenness on poor pregnancy outcomes showed a significant decrease of about 6% for LBW, VLBW, and SGA (pooled standardized odds ratio OR, 0.94; 95% CI, 0.92, 0.97) [20, 22, 28, 29, 39] (Fig. 5).

**Fig. 6:**
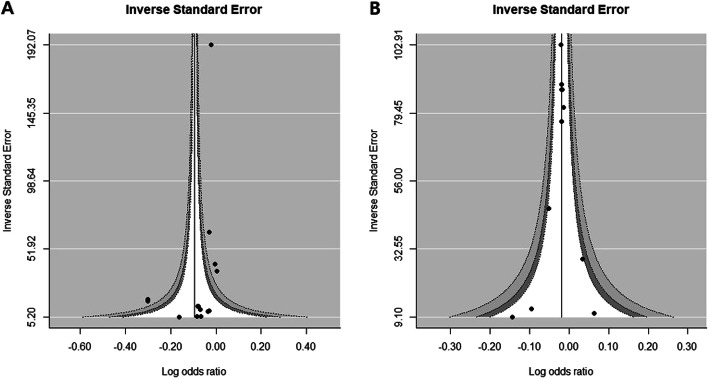
Funnel plot. The plot shows low birthweight related pregnancy outcomes (LBW, VLBW, or SGA) (**a**, *P* = 0.3556 for Egger’s test) and preterm birth (PTD or VPTD) (**b**, *P* = 0.6952 for Egger’s test). Abbreviations: LBW: low birthweight; VLBW: very low birthweight; SGA: small for gestational age; PTD: preterm delivery; VPTD: very preterm delivery

However, the overall NDVI effect of reducing poor pregnancy outcomes should be interpreted with caution as the subgroup results for each buffer size (100, 250, and 500 m) did not reach statistical significance. Nevertheless, low occurrences of LBW, VLBW, and SGA were shown at all NDVI distances. No publication biases were observed in the study adjusted for demographic and civilization factors (Fig. 6a) and poor pregnancy outcomes (LBW, VLBW, and SGA) were not statistically significant (Supplementary Figure S3 B).

**Fig. 7:**
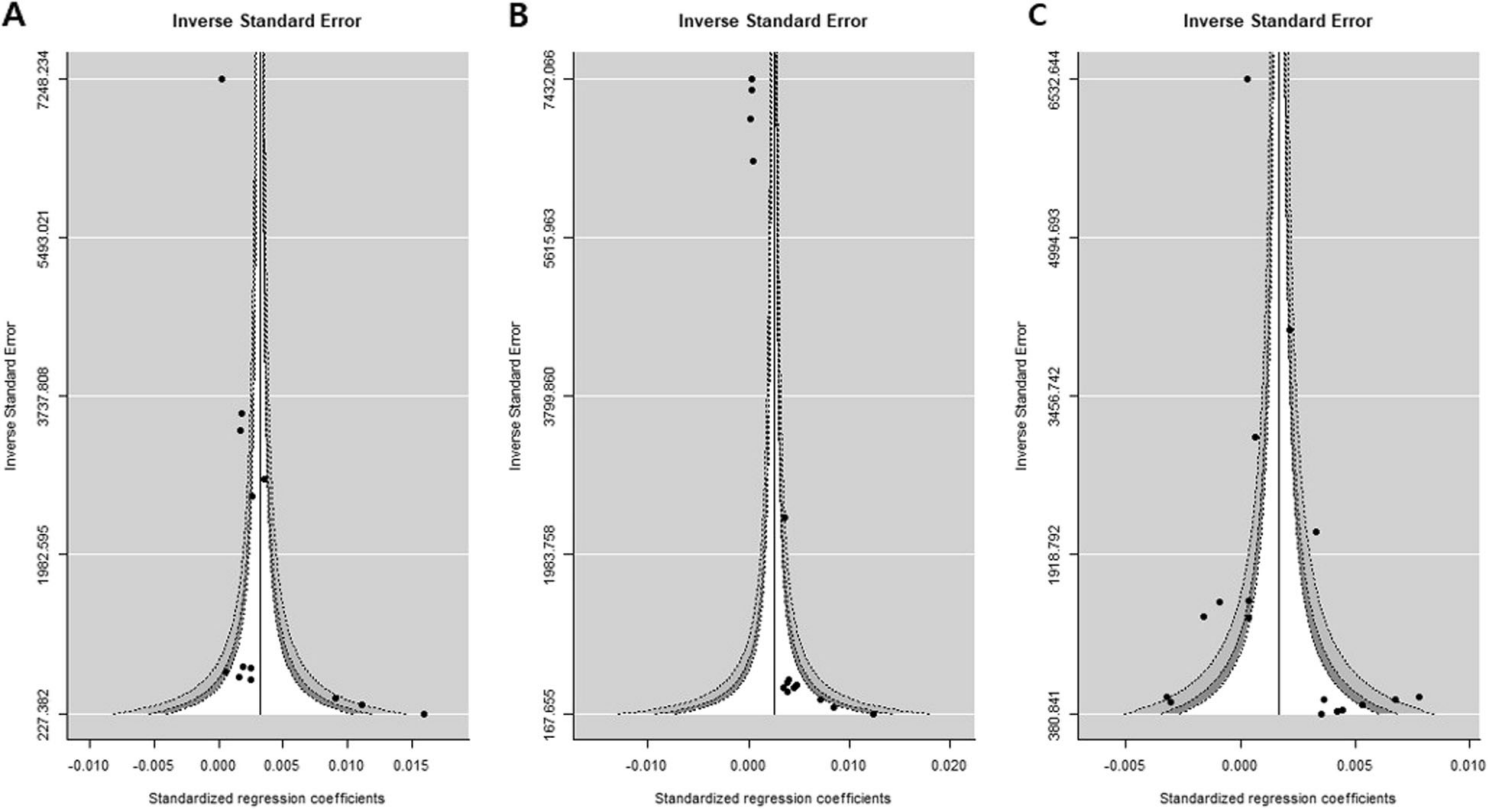
Funnel plots of term birth weight for publication bias. The analysis was adjusted for demographic characteristics of the subjects (left panel, *P* = 0.0005 for Egger’s asymmetry test of the funnel plot), with further adjustment for air quality factors (middle panel, *P* < 0.0001 for Egger’s test) and demographic and civilization factors regardless of air quality effects (right panel, *P* = 0.0881 for Egger’s test)

**Fig. 8:**
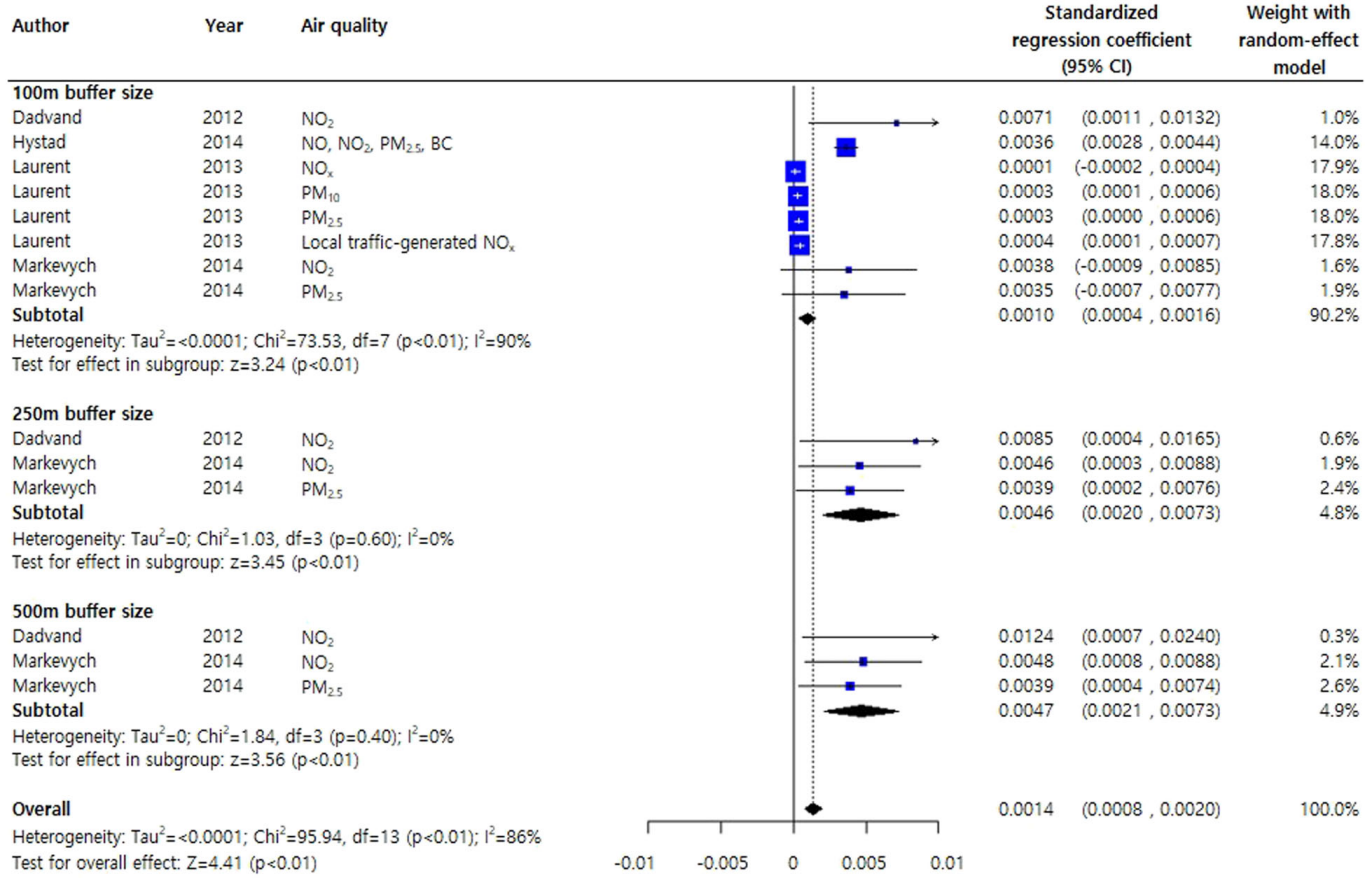
Meta-analysis of the standardized regression coefficients of the effects of greenness on term birth weight. Adjusted for subject demographic characteristics and air-quality–related factors

To evaluate publication bias, the contoured enhanced funnel plot for birthweight data was reviewed. Figure 7 shows no asymmetry within these plots, except for the middle panel. The middle panel funnel plot indicated some non-significant small effect-size studies lacking adjustment for demographic characteristics and air quality (Fig. 7b). There were small values for the inverse standard error of the regression coefficient for the NDVI effect, i.e., small sample sized studies found at the lower white space in the funnel plot. This possibility of publication bias partly explained the unexpectedly large NDVI effects associated with a buffer size of 250 and 500 m observed in Fig. 8.

A random-effects meta-regression was conducted to investigate changes in the standardized mean birthweight for different NDVI buffer sizes after adjusting for demographic and civilization factors but not air quality (Supplementary Figure S3A). As the buffer size increased, the variability of the standardized mean birthweight decreased (regardless of air quality), although the trend was not statistically significant (P for linear trend = 0.5157).

**Fig. 9:**
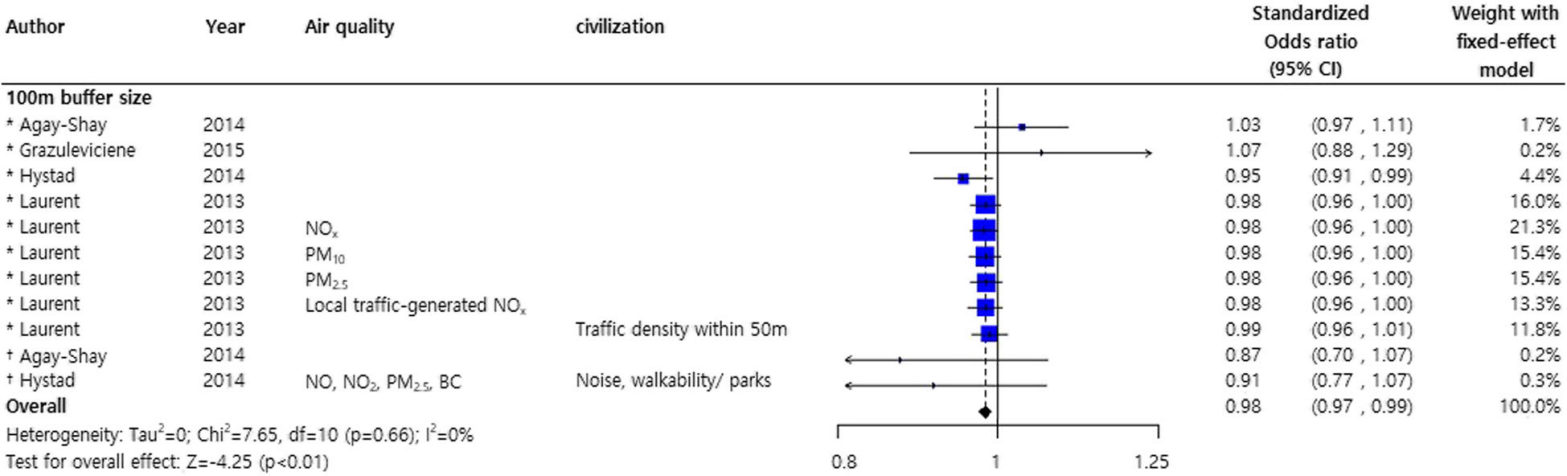
Forest plot of the effects of greenness on preterm delivery* or very preterm delivery†. Due to the limited number of studies, only the results for 100-m buffer size are pooled. Abbreviations: PTD: preterm delivery; VPTD: very preterm delivery

The meta-analysis of the effect of greenery on preterm birth included only a 100-m NDVI buffer [22, 25, 29, 35, 39]. A statistically significant 2% average decrease in preterm birth was observed (pooled OR 0.98; 95% CI, 0.97, 0.99) (Fig. 9) and no obvious publication bias was detected (Fig. 7b).

## Discussion

We performed a meta-analysis to investigate the effect of green and civil environments on the pregnancy outcomes while considering the associated factors, such as ambient air pollution and civilization. To the best of our knowledge, this is the first study to extend the work to assess the effects of greenness in with the context of the proximity to air pollution sources on the pregnancy outcomes. This study was provided stratification of greenness and adjusted air quality for the investigation of poor pregnancy outcomes, such as LBW, SGA, and preterm birth, showing a weak positive effect and a significant association based on the pooled standardized regression coefficient.

Numerous reports on greenness, including case studies and epidemiological and observational studies, have reported positive maternal and infant health outcomes. Those studies analyzed the effects of greenness measurements such as the NDVI. We cautiously interpreted our analyses with the restriction of the greenness effect such that it was measured by NDVI derived from the Landsat Enhanced Thematic Mapper Plus data at a resolution of 30 by 30 m. Therefore, we excluded several studies, including those that measured NDVI using Google Maps or Scotland’s Greenspace Map [26, 40–43]; those that adjusted for the effects of water-related variables to estimate the NDVI effect [44]; those with NDVI based on land surface reflectance of visible (red) and near-infrared wavelengths [45]; and those with NDVI and surface radiation temperature, which tended to have the lowest surface water values [46].

In this meta-analysis, the investigation of the association between greenness and pregnancy outcomes might assume that exposure to greenness would result in reduced stress and related distress, increased physical activity, and enhanced social participation [5, 6, 8, 47]. While weight gain during pregnancy is a normal process and related to a 50 to 70% decrease in insulin sensitivity compared to that in non-pregnant women, excessive weight gain is related to adverse maternal and neonatal outcomes [48–50] The few studies that investigated the health benefits of exercise for pregnant women reported no adverse pregnancy outcomes and a change in pregnant life toward a healthier and more active lifestyle [51]. Greenness has been associated with increased physical activity levels. The findings of our study revealed that increased NDVI was consistently positively correlated with birthweight. Furthermore, the dose-response relationship between NDVI buffer distance and birth outcome was examined in a meta-regression analysis revealed a negative trend but no significant difference. Comparisons of the birthweight results revealed a pooled standardized regression coefficient for the 100-m buffer distance adjusted for demographic characteristics and air quality of 0.001 (95% CI, 0.0004, 0.0016). This value did not differ from that obtained in the former meta-analysis; however, the statistical significance increased as the CI narrowed. If the interpretation of our results remains questionable, some uncertainties can be resolved by examining the findings of the civilization-adjusted effect size (0.0026, 95% CI: 0.0001, 0.0050 for the 100-m buffer distance) or larger buffer distance (0.0046 and 0.0047 for 250- and 500-m buffer distances, respectively).

Assessed according to NDVI buffer distance, the frequency of LBW, VLBW, or SGA was associated with low NDVI for all buffer sizes, although there were no significant relationships between NDVI buffer distance and poor fetal weight gain.

The environmental factors were challenging to measure or quantify; therefore, this study attempted to include adjusted summary measures of each study with standardized principles, investigating each demographic characteristic separately, including air quality and civilization. Moreover, considering air quality, adjustment for the concentrations of fine PM was analyzed separately to precisely identify the effects of this confounder (Supplementary Figure S2). The overall standardized regression coefficient was 0.0003 (95% CI: 0.0002, 0.0005), which showed the same direction but a smaller coefficient than that for the adjustment for overall air quality. Because the concentration of the PM itself has an adverse effect on birthweight [52, 53], it is important to adjust for this factor and provide a careful interpretation. Interestingly, increased greenness showed decreased PTD or VPTD despite poor air quality and civil environment (population density, traffic density, and noise, among others) Therefore, further research for the public health community regarding residential greenness related to environmental medicine and public health is needed to investigate the associations among residential greenness, air pollution, fetal development, and pregnancy outcomes.

Indeed, living in a green space is associated with positive impacts on the health and well-being of pregnant women. In Korea, a forest therapy called “Sup-TaeGyo” was specifically developed as a prenatal education program and provided some evidence for the physical and psychological benefits of exposure to greenness, including its impact on prenatal health and birth outcomes (Supplementary Table S2). Various antenatal programs are traditionally offered to support the mental and physical health of pregnant women over the whole pregnancy period to ensure a healthy birth. Lifestyle interventions with education or instruction on behaviors such as eating habits and physical activity during pregnancy have been emphasized to help prevent adverse pregnancy outcomes [54, 55]. Most of the reports were case studies written in the Korean language that evaluated mental health outcomes, mainly depression and emotional status, but which did not report physical health outcomes.

This study may be challenging to assemble an evidence base for the effect of greenness and associated air pollution on the birth outcomes; thus, more research is needed to validate and evaluate the health outcomes and to identify underlying mechanisms. Although air pollution and civilization were considered potential mediators in this study, other factors, such as physical activity and social interaction, may also affect the health outcomes. In the future, robust randomized clinical trials should be conducted to evaluate the effect of the greenness environment related to air pollution and civilization on diverse maternal health and birth outcomes. Systematic reviews and meta-analyses are needed to investigate the relationship between residential environments with various greenery and pregnancy outcomes with the categorization of maternal and fetal health to aid in the development of future guidelines for green space life.

This study provides evidence that a positive association between greenness and pregnancy outcomes has certain limits on the diversity of the interventions used. The greenness measurement methodologies associated with pregnancy outcomes has made some difficulties in the selection of eligible studies and might remain a barrier to further research. As a method of greenness measurement, the NDVI is not adequate for determining the types of plants and their uses, such as a private garden or public park.

An observational study included in this analysis focused on the effects of greenness during pregnancy and reported another limitation that the greenness properties do not affect the viability in the outcome process. Tracking the results from the time of conception or prior to conception will aid in addressing this limitation.

Table 1 shows the nearly 10-year gap between the dates of publication of previous studies and the present study. While no publication bias was observed, the research findings (pregnancy outcomes) should be interpreted cautiously in consideration of the development of medical technology, changes in the greenness of the environments, and development of urban civilizations in this time period.

## Conclusions

The results of this review suggest that the greenness of the environment has a positive effect on pregnancy outcomes including decreased LBW, SGA, or preterm birth, despite poor air quality and civilization. However, some of the studies included in the meta-analyses had poor study quality and high heterogeneity; thus, researchers should investigate further with caution. In this context, in the era of low fertility and aging, emphasis should be placed on increasing the social agreement for including green spaces in cities, the importance of green space management and planning in terms of environmental medicine, and public health.

## Supplementary information

**Additional file 1 **: **Supplementary Figure S1.** Sensitivity analysis of the effects of greenness on term birth weight adjusted for subject demographic characteristics only by omitting the most influential study result, as described by Dadvand et al. (2012c) [16]

**Additional file 2 **: **Supplementary Figure S2**. Sensitivity analysis of the effects of greenness on term birth weight, adjusted for subject demographics and PM_x_ as a measure of air pollution

**Additional file 3 **: **Supplementary Figure S3.** A random-effects meta-regression analysis of buffer sizes on the standardized regression coefficient for birth weight (A) and on the standardized logarithm of low birthweight (LBW, VLBW, or SGA) per 0.1 NDVI meter increases (B), both adjusted for subject demographic variables and civilization factors. Abbreviations: LBW: low birth weight; VLBW: very low birth weight; SGA: small for gestational age; NDVI: normalized difference vegetation index

**Additional file 4 **: **Supplementary Table S1**. Search terms used for the review. **Supplementary Table S2**. Studies on antenatal education in green space in Korea that investigated “Sup-TaeGyo”.

## Data Availability

The datasets generated and analyzed during the current study are not publicly available due to lack of permission but are available from the corresponding author on reasonable request.

## Abbreviations

NDVI: Normalized difference vegetation index
ETM +: Enhanced Thematic Mapper Plus
LBW: Low birth weight
VLBW: Very low birth weight
SGA: Small for gestational age
PTD: Preterm delivery
VPTD: Very preterm delivery
OR: Odds Ratio
SD: Standard deviation
CI: Confidence interval
IQR: Interquartile range
PM: Particular matter
RoBANS: Risk of Bias Assessment tool for Non-randomized Study
