# Supplementary figures and images for: Greenness, civil environment, and pregnancy outcomes: perspectives with a systematic review and meta-analysis
Source: Environ Health. 2020 Aug 27;19:91. doi: 10.1186/s12940-020-00649-z (PMC7457282; doi:10.1186/s12940-020-00649-z)

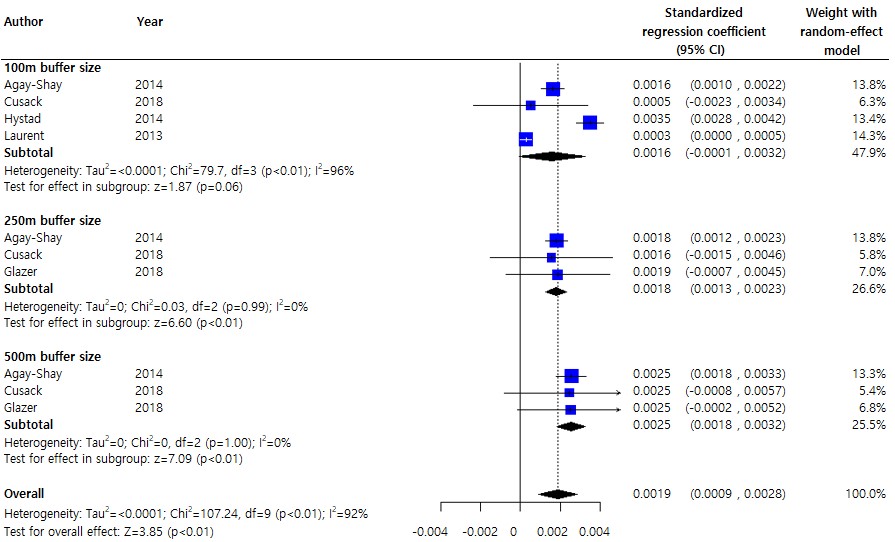

Supplement: Supplementary file 1 — Additional file 1 : Supplementary Figure S1. Sensitivity analysis of the effects of greenness on term birth weight adjusted for subject demographic characteristics only by omitting the most influential study result, as described by Dadvand et al. (2012c) [16] [file 12940_2020_649_MOESM1_ESM.jpg]

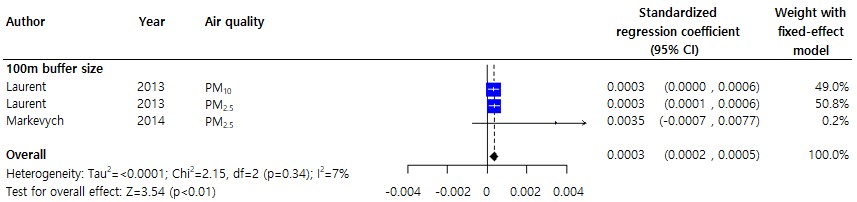

Supplement: Supplementary file 2 — Additional file 2 : Supplementary Figure S2. Sensitivity analysis of the effects of greenness on term birth weight, adjusted for subject demographics and PMx as a measure of air pollution [file 12940_2020_649_MOESM2_ESM.jpg]

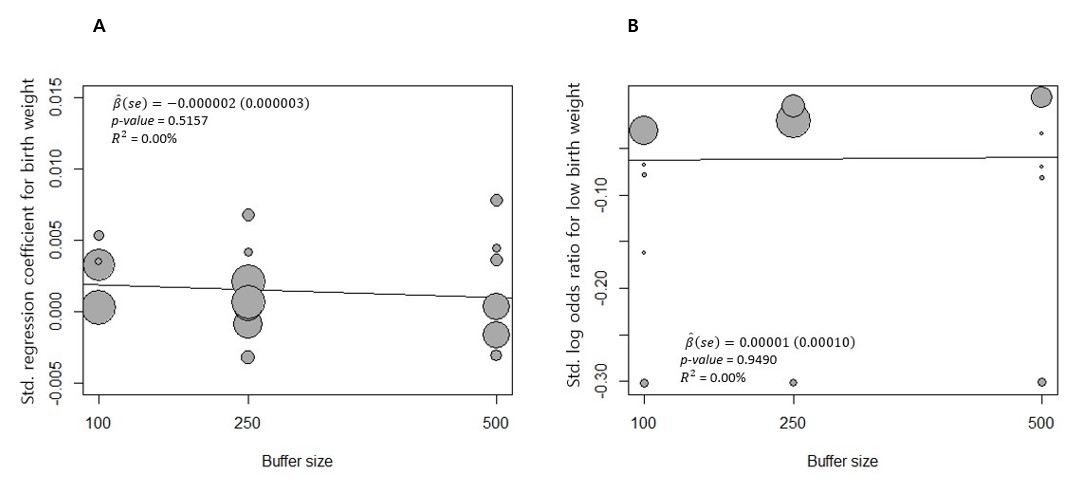

Supplement: Supplementary file 3 — Additional file 3 : Supplementary Figure S3. A random-effects meta-regression analysis of buffer sizes on the standardized regression coefficient for birth weight (A) and on the standardized logarithm of low birthweight (LBW, VLBW, or SGA) per 0.1 NDVI meter increases (B), both adjusted for subject demographic variables and civilization factors. Abbreviations: LBW: low birth weight; VLBW: very low birth weight; SGA: small for gestational age; NDVI: normalized difference vegetation index [file 12940_2020_649_MOESM3_ESM.jpg]
