# Supplementary material for: Greenness, civil environment, and pregnancy outcomes: perspectives with a systematic review and meta-analysis
Source: Environ Health. 2020 Aug 27;19:91. doi: 10.1186/s12940-020-00649-z (PMC7457282; doi:10.1186/s12940-020-00649-z)
Supplement: Supplementary file 4 — Additional file 4 : Supplementary Table S1. Search terms used for the review. Supplementary Table S2. Studies on antenatal education in green space in Korea that investigated “Sup-TaeGyo”. [file 12940_2020_649_MOESM4_ESM.docx]

**Supplementary Table S1**. Search terms used for the review

|  | Keywords | Mesh/Entree | Free-text (title, abstract, keyword) |
| --- | --- | --- | --- |
| Population | Pregnant woman | Pregnant Women | Pregnant Women |
|  | Pregnancy | Pregnancy | Pregnancy |
| Intervention | Green space |  | Green space(s)  Greenspace(s) |
|  | Parks | Parks, Recreational | Parks, Recreational |
|  | Forest(s) | Forests | Forest(s) |
|  | greenness |  | Greenness |
| Outcome | Pregnancy outcome(s) |  | pregnancy outcome(s) |
|  | Birth outcome(s) |  | birth outcome(s) |

**Supplementary Table S2**. Studies on antenatal education in green space in Korea that investigated “Sup-TaeGyo”

| First author (year) | Title | Study design | Study population (N) | Duration of intervention | Pregnancy Outcome | Published in Korea |
| --- | --- | --- | --- | --- | --- | --- |
| Lee Mi-Na (2014) | The Development and Effects of Forest Prenatal Education using Therapy of Āyurveda | Case control study | N = 30  Pregnant women  with their husbands | 10 Sessions  (2 h Each) | Parental identity,  Marital intimacy,  Parental-fetus attachment | Doctoral thesis |
| Song et al. (2009) | The Influence of Forest Therapeutic Program on Unmarried Mothers’ Depression and Self-Esteem | Case control study | N = 70 Unmarried pregnant women and mothers | 8 Sessions x 3 times (Each time 2 nights and 3 days) | Depression, Self-esteem | Journal of Korean Forest society |
| Jang Sun-Hee. (2015) | Effects of Forest Prenatal Education Program on Stress and Emotional Stability of Pregnant Women | Case control study | N = 101 | 8 Sessions (Each time 2 nights and 3 days) | Pregnancy Stress, Emotional Stability | Master’s thesis |
| Park et al. (2011) | The Effects of The Forest Prenatal Program Using Emotional Rest and attachment of Pregnant Women | Case control study | N = 100 | 4 sessions (Once a week) | Emotional evaluation, Maternal identity, SEQ(Session Evaluation Questionnaire) | Korean Institute Of Forest Recreation and Welfare |
| Lee et al. (2015) | The Effects of Prenatal Education in Forest on the Mindfulness and Psychological Well-Being of Pregnant Woman | Case control study | N = 14 | 8 sessions (Each time 90 min) | Current awareness, attention of mindfulness, acceptance judgmental criticism and de-centered care | Journal of the Korean Institute of Forest Recreation |
| Kim et al. (2012) | The Stress Reduction Effects of Forest Prenatal Education on Pregnant Woman | Case control study | N = 51 | 2 sessions (1 night and 2 days) | Psychological change, physical change | Journal of Forest Science |
